# Supplementary material for: The genome sequence of the short-beaked common dolphin, Delphinus delphis Linnaeus, 1758
Source: Wellcome Open Res. 2025 Apr 8;10:178. [Version 1] doi: 10.12688/wellcomeopenres.23918.1 (PMC12022549; doi:10.12688/wellcomeopenres.23918.1)
Supplement: Peer review history

# Reviewer report: Reviewer response for version 1

**DOI:** 10.21956/wellcomeopenres.26387.r122095

**Published:** 2025-04-24

**Authors:** Annabel Whibley

**Affiliations:** 1 Grapevine Improvement, Bragato Research Institute, Lincoln, New Zealand

Davidson and Morin report the generation of a reference genome assembly for the short-beaked common dolphin, *Delphinus delphis*. PacBio Hifi  (assembly backbone) and Illumina HiC (scaffolding) reads contribute to the assembly. RNaseq data was also generated and an Ensembl genome annotation has also been produced. The report follows the DToLtemplating, with comprehensive and accurate metadata, appropriate wet lab and bioinformatics workflows and well-structured reporting of metrics. Public links are active.

 The coverage of the PacBio reads is <30x  and perhaps a bit marginal- whilst completeness of the assembly is excellent, the assembly is quite fragmented and with perhaps a little more retained duplication than would be expected. The Y chromosome scaffold is 4Mb:  this seems unusually small to me and I wonder if it is only partially resolved in this dataset?  Overall the genome assembly is of high quality (6.8.Q66 ) and will serve as a valuable resource for the community.

Are sufficient details of methods and materials provided to allow replication by others?

Yes

Is the rationale for creating the dataset(s) clearly described?

Yes

Are the datasets clearly presented in a useable and accessible format?

Yes

Are the protocols appropriate and is the work technically sound?

Yes

Reviewer Expertise:

Genomics Bioinformatics

I confirm that I have read this submission and believe that I have an appropriate level of expertise to confirm that it is of an acceptable scientific standard.

# Reviewer report: Reviewer response for version 1

**DOI:** 10.21956/wellcomeopenres.26387.r122097

**Published:** 2025-04-16

**Authors:** Takushi Kishida

**Affiliations:** 1 Nihon University, Fujisawa, Japan

This study provides a high-quality chromosomal-level *Delphinus delphis* haploid genome assembly. I have no complaints about the quality of this genome data.

 I hope, if possible, the authors deposit the specimen used for genome sequencing in an appropriate museum so that a vouchered specimen associated with this genome data is available.

Are sufficient details of methods and materials provided to allow replication by others?

Yes

Is the rationale for creating the dataset(s) clearly described?

Yes

Are the datasets clearly presented in a useable and accessible format?

Yes

Are the protocols appropriate and is the work technically sound?

Yes

Reviewer Expertise:

evolutionary genomics, population genetics

I confirm that I have read this submission and believe that I have an appropriate level of expertise to confirm that it is of an acceptable scientific standard.
